# Supplementary material for: Deep optoacoustic localization microangiography of ischemic stroke in mice
Source: Nat Commun. 2023 Jun 16;14:3584. doi: 10.1038/s41467-023-39069-1 (PMC10275987; doi:10.1038/s41467-023-39069-1)
Supplement: Supplementary file 1 — Supplementary Information [file 41467_2023_39069_MOESM1_ESM.pdf]

SUPPLEMENTARY INFORMATION

Supplementary Note 1. Absorbed energy in a highly-absorbing sphere.

Supplementary Note 2. Estimation of light fluence from polydisperse particles.

Supplementary Note 3. Estimation of dichloromethane and IR-780 concentration in the emulsion.

Supplementary Figure 1. Effect of light attenuation in the absorbed optical energy within the microdroplets.

Supplementary Figure 2. Imaging performance as a function of number of frames.

Supplementary Figure 3. Fluence estimation with monodisperse and polydisperse particles.

Supplementary Figure 4. Localization optoacoustic tomography (LOT) images for n=5 mice taken after stroke.

Supplementary Figure 5. Localization optoacoustic tomography (LOT) images and velocity maps of a mouse taken before and after stroke.

Supplementary Figure 6. Enhancement of penetrating vessels via Sobel filtering.

Supplementary Figure 7. Velocity maps for n=5 mice taken after stroke.

Supplementary Figure 8. Bio-distribution of oxygenated and deoxygenated hemoglobin for n=5 mice after stroke.

Supplementary Figure 9. Numerical simulation of errors in oxygen saturation readings for neighboring microvessels.

Supplementary Figure 10. Cortical perfusion assessment with laser speckle contrast imaging (LSCI).

Supplementary Figure 11. Blood flow velocity quantification after microdroplet injection.

Supplementary Figure 12. H&E-stained histological images of major organs.

Supplementary Figure 13. Signal-to-noise ratio (SNR) of optoacoustic (OA) and pulse-echo ultrasound (US) images of microdroplets and microbubbles.

Supplementary Figure 14. Concentration of droplets as a function of time post injection.

Supplementary Figure 15. Point spread function of the optoacoustic tomography (OAT) system.

Supplementary Figure 16. Concentration of dichloromethane and IR-780.

Supplementary Note 1. Absorbed energy in a highly-absorbing sphere.

For a light-absorbing microsphere with diameter in the order of a few microns, the light fluence is assumed to be constant within its volume. Thereby, the absorbed energy is considered to be proportional to the volume of the sphere. However, strong light absorption within the microdroplets can result in strong light attenuation that needs to be taken into account to estimate the absorbed energy.

In a light-scattering medium such as a biological tissue, the light radiance is assumed to be isotropic. Considering no transient effects and no light sources inside the medium, the light fluence ($U(\vec{r})$, energy per unit area) obeys the following equation [1]

$$-D\nabla^2 U(\vec{r}) + \mu_a U(\vec{r}) = 0, \qquad (1)$$

where $D = (1/3)/(\mu_s' + \mu_a)$, being μ_a and μ_s' the absorption and reduced scattering coefficients, respectively. Considering spherical symmetry for the light being absorbed in a microsphere, Eq. 1 can be expressed as

$$r^2 \frac{\partial^2 U(r)}{\partial r^2} + 2r \frac{\partial U(r)}{\partial r} - k^2 r^2 U(r) = 0, \qquad (2)$$

where r is the distance to the center of the sphere and $k = \sqrt{\mu_a/D}$. The general solution of Eq. 2 is given by [2]

$$U(r) = A\sqrt{\frac{\pi}{2kr}} I_{1/2}(kr) + B\sqrt{\frac{\pi}{2kr}} I_{-1/2}(kr), \qquad (3)$$

where $I_n(\ \ )$ are modified spherical Bessel functions of the first kind and A and B are two arbitrary constants. Considering as boundary conditions $U(0) \neq \infty$ and $U(R) = U_0$, being R the radius of the microsphere, Eq. 3 can be expressed as

$$U(r) = U_0 \frac{R\sinh(kr)}{r\sinh(kR)}. \qquad (4)$$

The absorbed energy in a shell of radius r can then be expressed as

$$dE(r) = U(r)\mu_a 4\pi r^2 dr = U_0 \mu_a \frac{R\sinh(kr)}{\sinh(kR)} 4\pi r dr. \qquad (5)$$

The total energy absorbed in the sphere is then calculated as

$$E = \int_0^R dE(r), \tag{6}$$

which results in

$$E = U_0\mu_a \frac{4\pi R^2}{k}\left[\coth(kR) - \frac{1}{kR}\right]. \tag{7}$$

Note that for a relatively low absorption coefficient $kR \ll 1$, in which case $\coth(kR)$ can be approximated as

$$\coth(kR) \approx \frac{1}{kR} + \frac{kR}{3}, \tag{8}$$

which results in

$$E_{\mathrm{unif}} \approx U_0\mu_a \frac{4}{3}\pi R^3, \tag{9}$$

Eq. 9 corresponds to the product of the fluence at the microsphere surface and the microsphere volume, i.e., the absorbed energy in the microsphere E_{unif} is estimated by considering a uniform fluence within the microsphere.

Considering a radius $R \approx 2.75\mu\mathrm{m}$ and an absorption coefficient $\mu_a \approx 50000\mathrm{cm}^{-1}$ (~200 mM of IR-780 dye with extinction coefficient ~$250\cdot 10^3$ $\mathrm{M}^{-1}\mathrm{cm}^{-1}$) for the dichloromethane microdroplets, the ratio $E/E_{\mathrm{unif}} \approx 0.12$. The value of this ratio as a function of the absorption coefficient is given in Suppl. Fig. 1.

The absorption coefficient within red blood cells is $\mu_a \approx 5\mathrm{cm}^{-1}$ (~5 mM of hemoglobin with extinction coefficient ~10^3 $\mathrm{M}^{-1}\mathrm{cm}^{-1}$). Thereby, E_{unif} is four orders of magnitude higher in the microdroplets. Considering the correction factor E/E_{unif} accounting for light attenuation, the absorbed energy within the droplets is three to four orders of magnitude higher than in red blood cells, depending on the relative volumes.

Supplementary Note 2. Estimation of light fluence from polydisperse particles.

The OA image intensity at the location of a microdroplet is directly proportional the optical energy absorbed within such microdroplet given by Eq. 7. For monodisperse (equal diameter) droplets, this is proportional to the local light fluence, while changes in size of individual microdroplets have an additional influence on the generated signals.

It is however possible to estimate the light fluence by considering the OA image intensities at the location of polydisperse microdroplets. To illustrate this, we consider as a representative example a semi-infinite medium with a typical effective attenuation coefficient $\mu_{\text{eff}} = 2.5\text{cm}^{-1}$ of biological tissues for near-infrared wavelengths. The light fluence distribution within this medium is given by [1]

$$U(z) = U_0 e^{-\mu_{\text{eff}} z}, \quad (10)$$

where z is the direction perpendicular to the surface.

We considered droplets with random diameters according to a distribution given by the histogram in Fig. 1b. An absorption coefficient $\mu_a = 50000\text{cm}^{-1}$ was considered for all droplets, which were uniformly randomly distributed across a field of view of 10x10 mm^2. For each droplet, the absorbed energy was calculated with Eq. 7 considering the light fluence at its position given by Eq. 10. The light fluence within the medium was estimated by least square fitting the estimated OA signals generated by the droplets to an exponential function. For comparison, the same fitting was also done for the OA signals generated by monodisperse microdroplets located at the same positions, estimated by considering a diameter of 5.5 μm. The estimated fluence as a function of number of polydisperse and monodisperse droplets is shown in Suppl. Fig. 3. The estimated attenuation coefficient is also plotted as a function of the number of monodisperse or polydisperse droplets considered.

Supplementary Note 3. Estimation of dichloromethane and IR-780 concentration in the emulsion.

Potential toxic effects of the microdroplets represent an important concern for the applicability of LOT in preclinical studies and for its eventual clinical translation. The total amounts of dichloromethane (DCM) and IR-780 to prepare the emulsion are ~16.6 g/l and ~1.6 g/l, respectively. Note, however, that the emulsion is filtered after vortexing, resulting in elimination of large droplets arguably accounting for most of the DCM and IR-780. Optical microscopy images showed that the concentration of droplets in the filtered emulsion is approximately 70 μl/l (Suppl. Fig. 16a), which corresponds to concentrations of DCM and IR-780 of ~93 mg/l and ~9 mg/l, respectively. In addition, the amount of IR-780 in the emulsion was quantified spectrophotometrically. For this, the absorption spectrum of a reference solution of IR-780 in ethanol was compared to that of the emulsion diluted in ethanol (Suppl. Fig. 16b). The estimated concentration of IR-780 obtained by this procedure was 0.18 g/l, indicating that the filtration has removed about 90% of the initial amount of the dye from the suspension. Assuming that the relative proportions of DCM and IR-780 are maintained, the estimated concentration of DCM in the emulsion is ~1.9 g/l. With an injection volume of 100 μl per 20 g body weight, the amounts of DCM and IR-780 injected in the animals (mice) are ~9.5 mg/kg and ~0.9 mg/kg, respectively. The DCM exposure limit in the workplace environment is set to 177 mg per m^3 of air [3]. This is based on the estimate that this exposure leads to less than 3% hemoglobin occupancy with carbon monoxide, the metabolic product of DCM, i.e., the maximum allowable uptake per day is ~14 mg/kg (assuming 70 kg of body weight). Note that DCM was also tested in the 1920s to maintain a person under anesthesia (~50 g DCM for 3 hours) [4], i.e., direct intravenous injection of sufficient amounts might have anaesthetic effects, yet the amounts used in the present study are

unlikely to be effective. Note, however, that while these calculations show that LOT can be successfully performed using non-toxic concentrations of DCM circulating in the blood, comprehensive toxicity tests are required for the eventual use of these droplets in humans. Note also that LOT imaging can still be performed by reducing the concentration of droplets (Suppl. Fig. 2). On the other hand, the injected concentration of IR-780 is below the reported toxicity in mice [5].

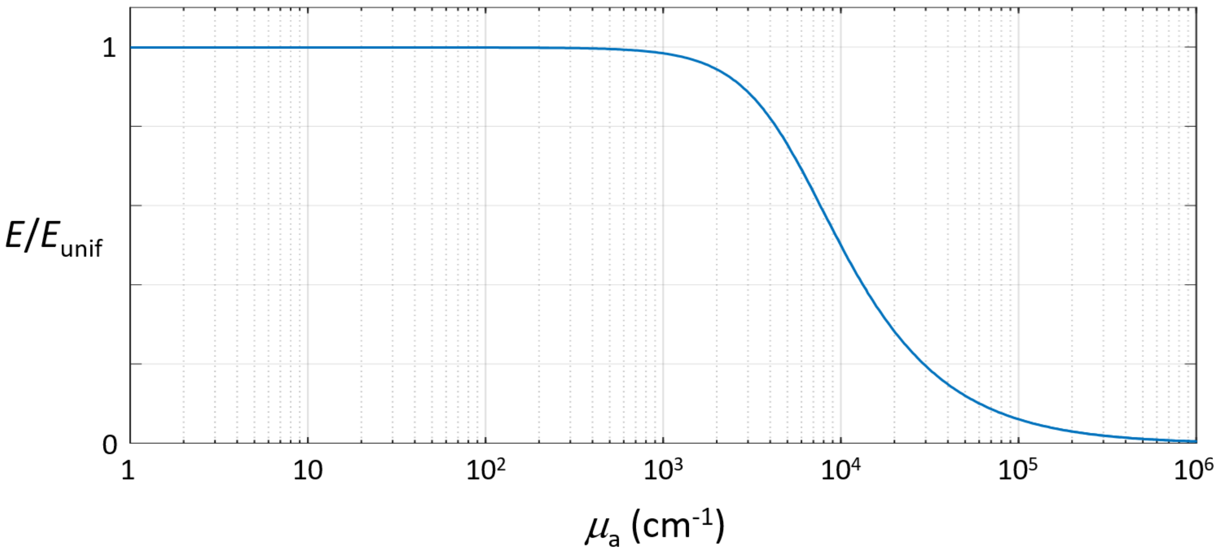

Supplementary Figure 1. Effect of light attenuation in the absorbed optical energy within the microdroplets. The absorbed optical energy E within a microdroplet with radius R=2.75 μm relative to the absorbed optical energy in the same microdroplet assuming uniform light fluence E_{unif} is shown. Details on the calculations are available in Suppl. Note 1.

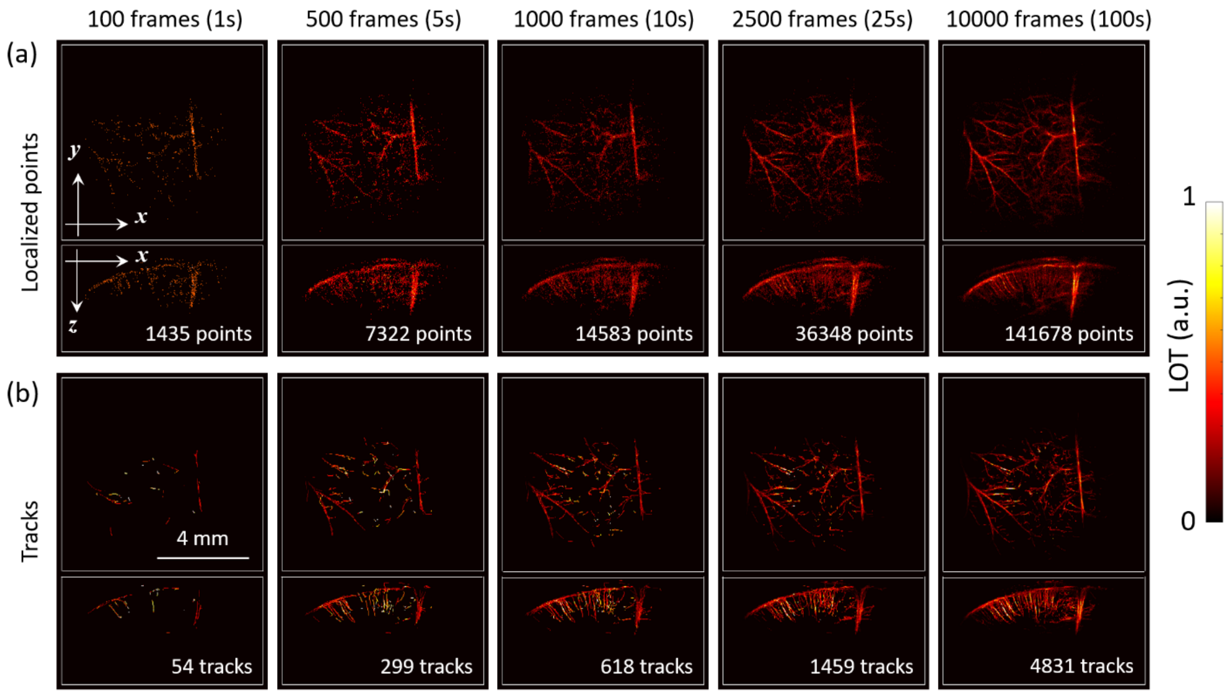

Supplementary Figure 2. Imaging performance as a function of number of frames. (a) Localization optoacoustic tomography (LOT) built by superimposing the localized points. (b) LOT image built by superimposing the tracks. Number of frames and corresponding recording time in seconds are indicated.

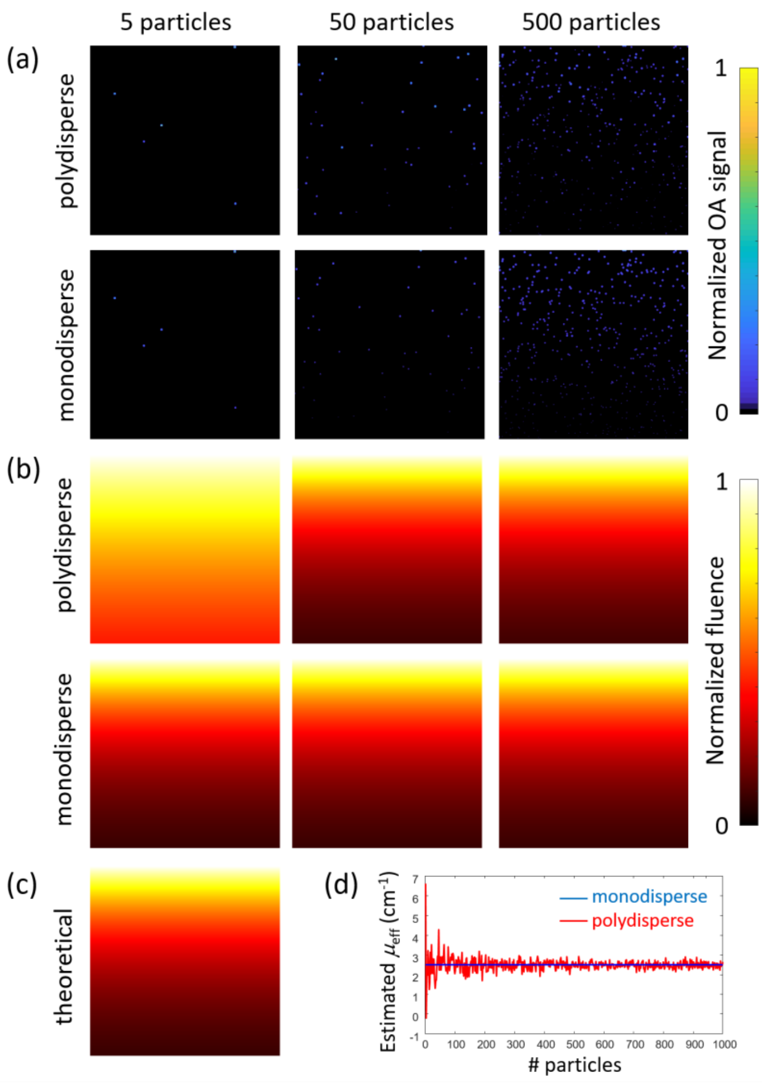

Supplementary Figure 3. Fluence estimation with monodisperse and polydisperse particles. (a) OA signals for randomly distributed particles. Top - polydisperse particles according to the measured size distribution. Bottom – monodisperse particles. Field of view – 10x10 mm^2. (b) Estimated fluence with the OA signals for the particles in (a). Top – polydisperse particles. Bottom – monodisperse particles. (c) Theoretical fluence used to calculate the OA signals. (d) Estimated effective attenuation coefficient μ_{eff} as a function of the number of monodisperse and polydisperse particles considered (randomly distributed). Details on the calculations are available in Suppl. Note 2.

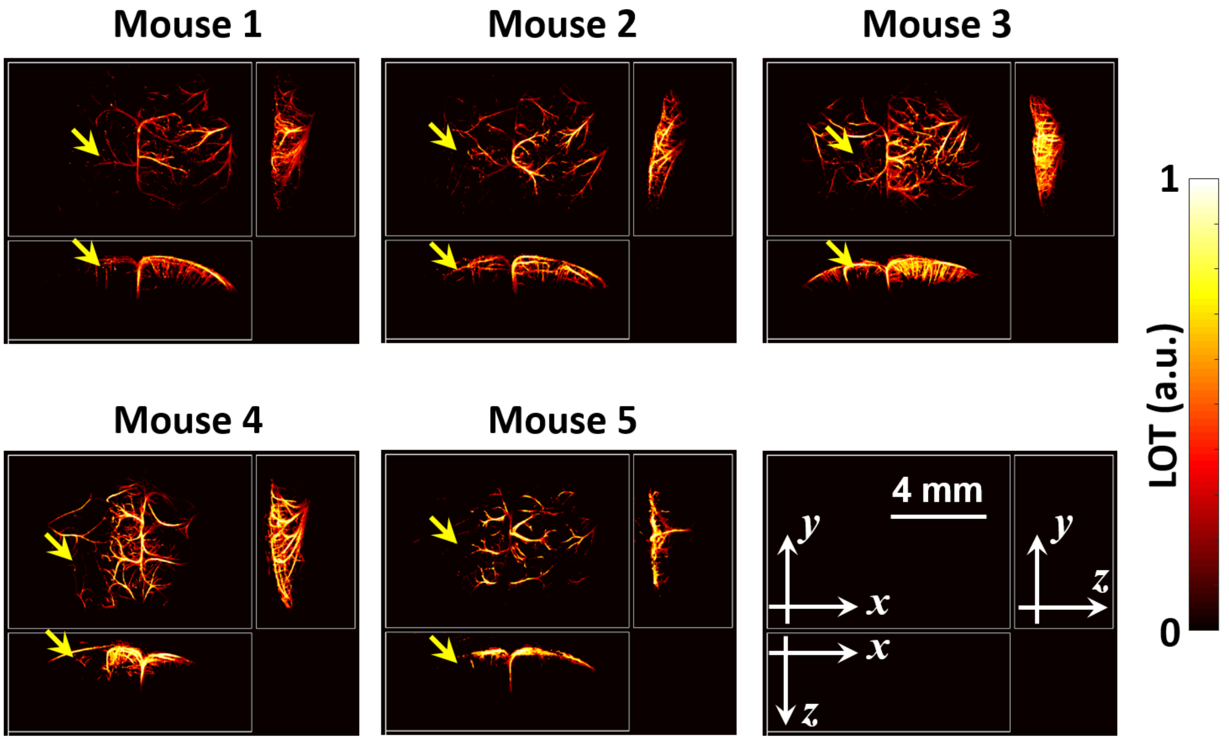

Supplementary Figure 4. Localization optoacoustic tomography (LOT) images for n=5 mice taken after stroke. Maximum intensity projections (MIPs) along the three Cartesian coordinates are shown. The areas affected by stroke is indicated with yellow arrows.

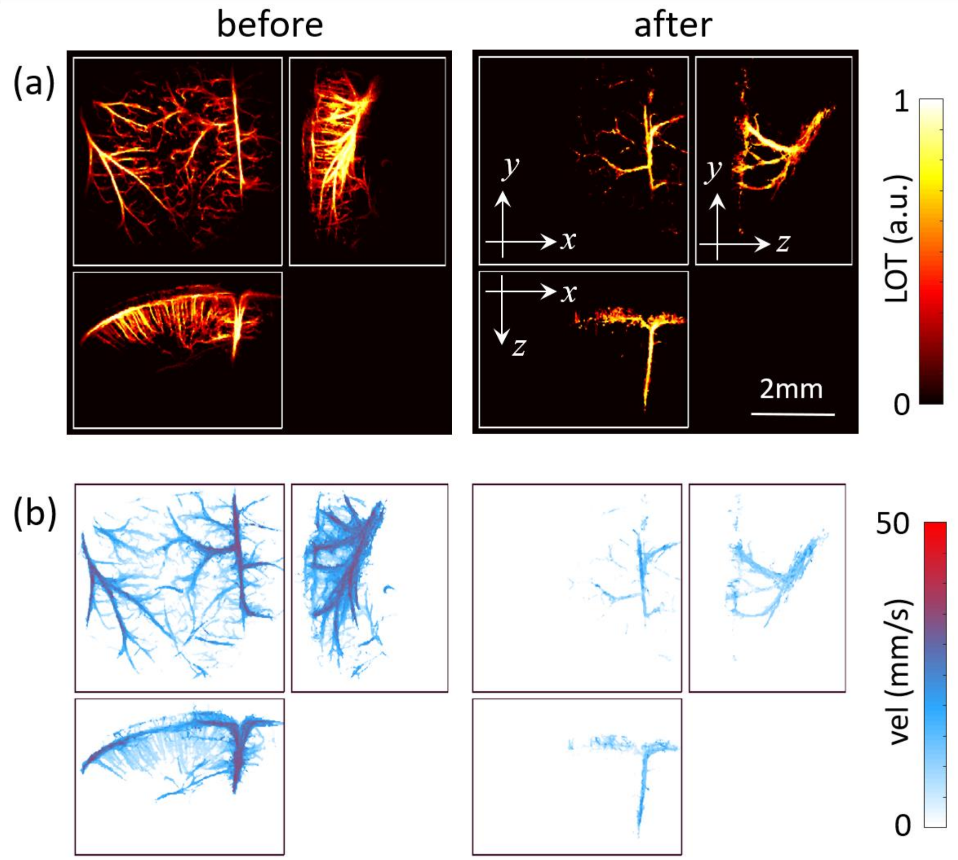

Supplementary Figure 5. (a) Localization optoacoustic tomography (LOT) images of a mouse taken before (left) and after (right) stroke. (b) Corresponding velocity maps. Maximum intensity projections (MIPs) along the three Cartesian coordinates are shown.

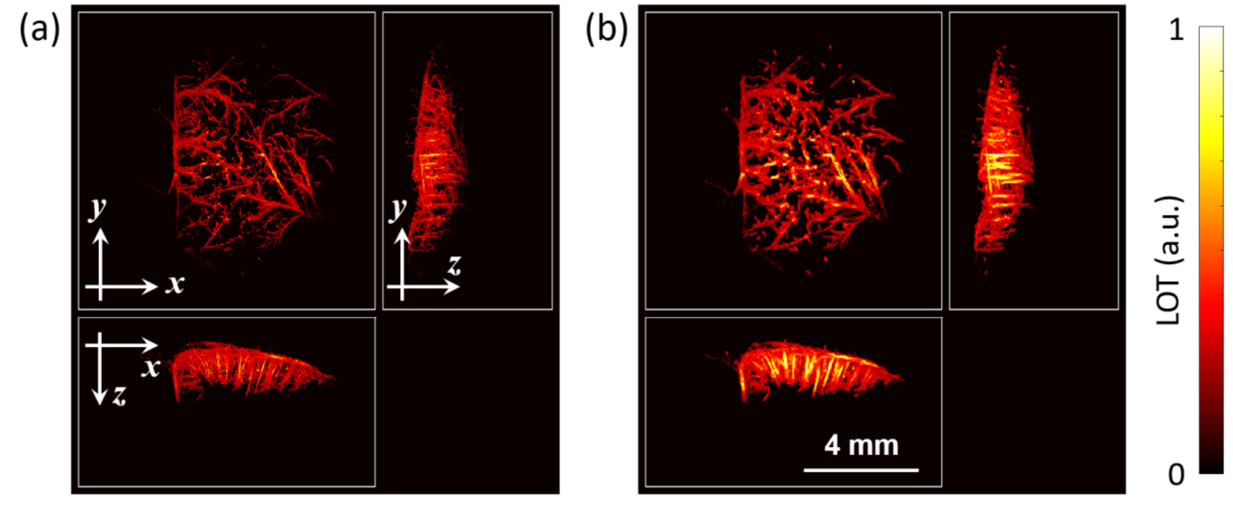

Supplementary Figure 6. Enhancement of penetrating vessels via Sobel filtering. (a) Original LOT image. (b) LOT image after application of a Sobel filter highlighting the gradient in the x-y plane. Maximum intensity projections (MIPs) along the three Cartesian coordinates are shown. Details are given in the methods section.

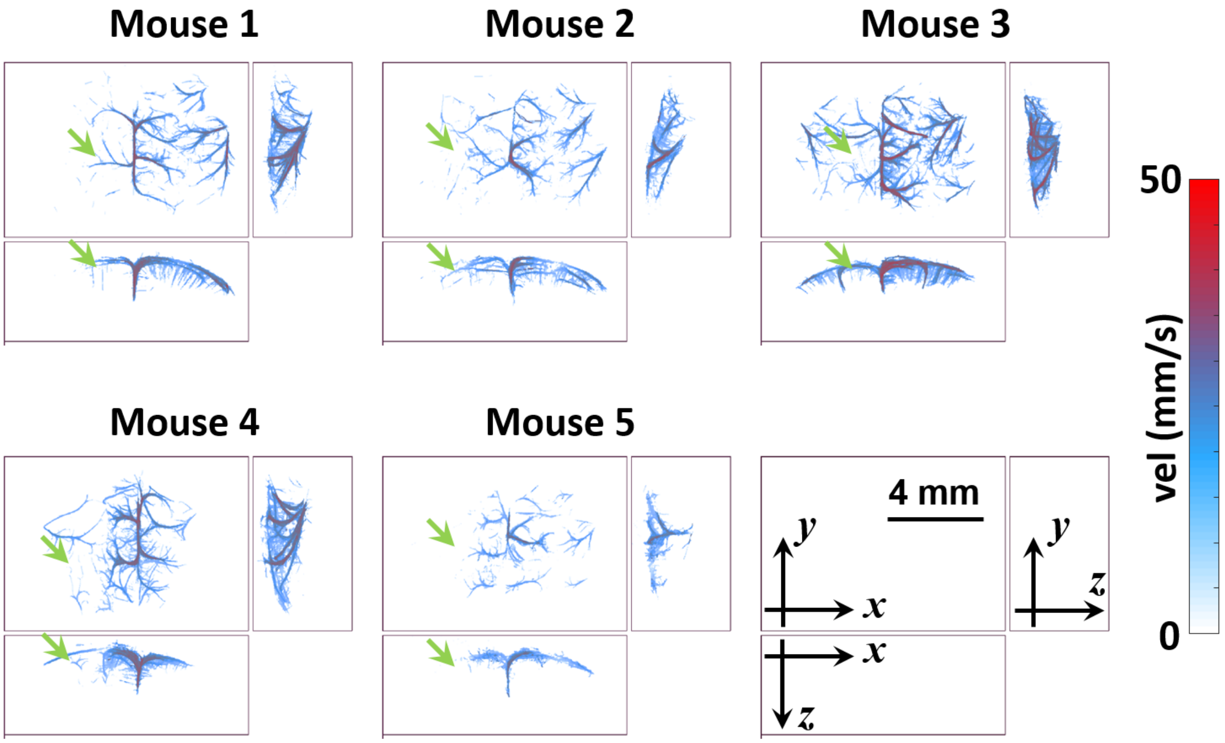

Supplementary Figure 7. Velocity maps for n=5 mice taken after stroke. Maximum intensity projections (MIPs) along the three Cartesian coordinates are shown. The areas affected by stroke are indicated with green arrows.

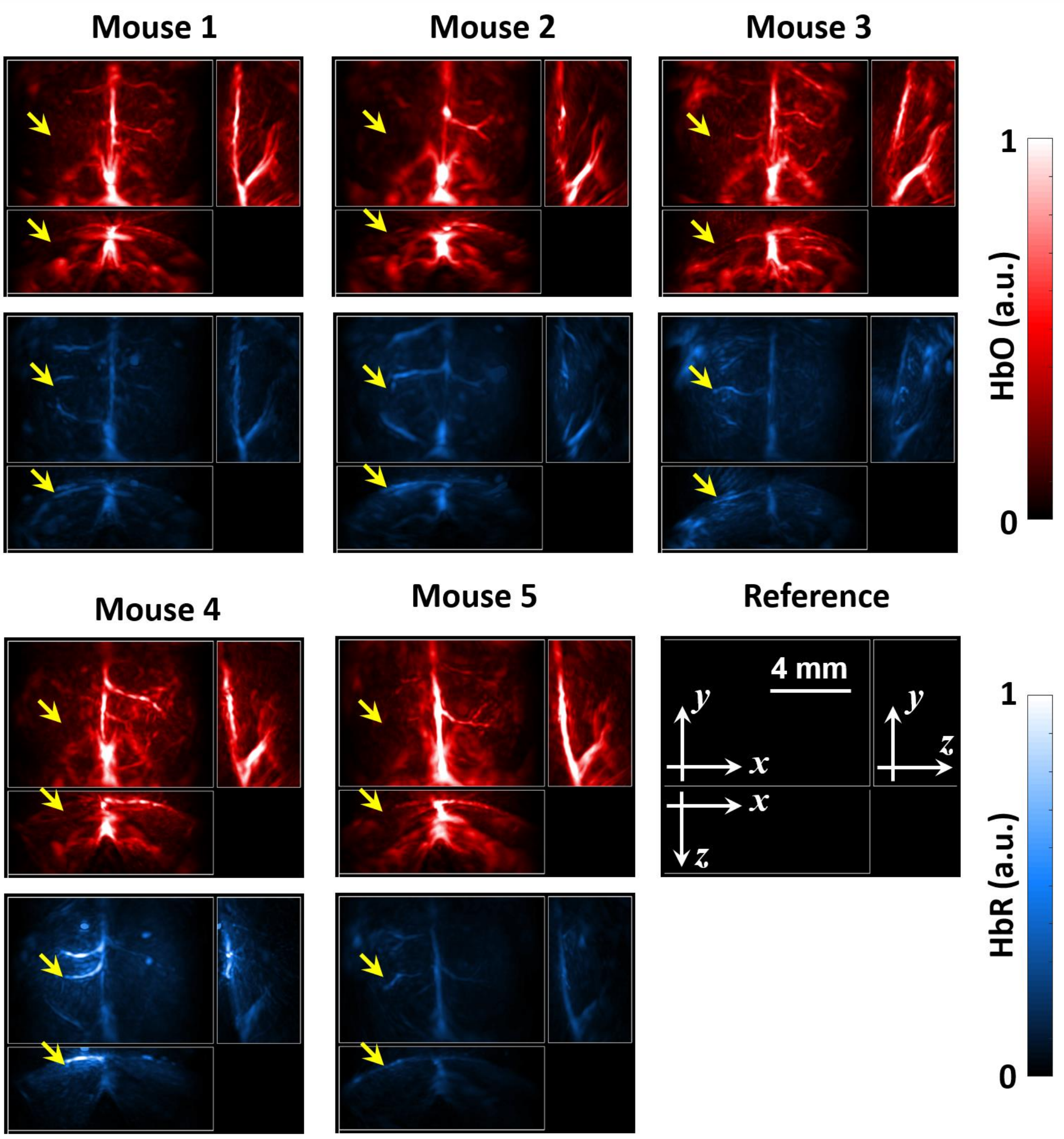

Supplementary Figure 8. Bio-distribution of oxygenated and deoxygenated hemoglobin for n=5 mice after stroke. Maximum intensity projections (MIPs) along the three Cartesian coordinates are shown. The areas affected by stroke are indicated with yellow arrows.

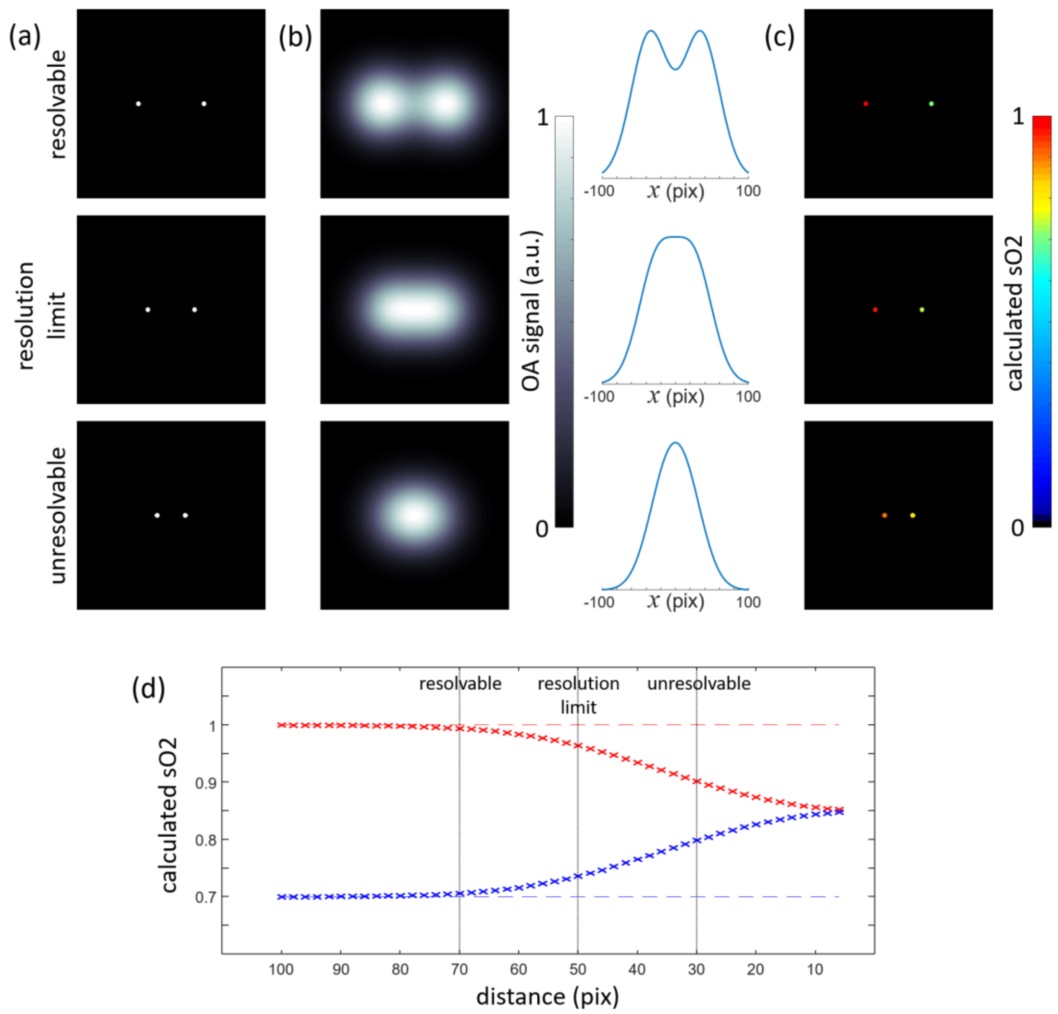

Supplementary Figure 9. Numerical simulation of errors in oxygen saturation readings for neighboring microvessels. (a) Cross-sectional images of two neighboring vessels with oxygen saturation levels of 100% and 70% mimicking an artery and a vein, respectively. (b) Images in (a) after convolution with a Gaussian function with full width at half maximum 25 pixels along with the central profiles of these images. (c) Oxygen saturation values calculated by considering the images in (b) by considering the images in (a) as a mask. (d) Calculated oxygen saturation values with this approach as a function of the separation between the vessels. Reference values are indicated as dashed lines.

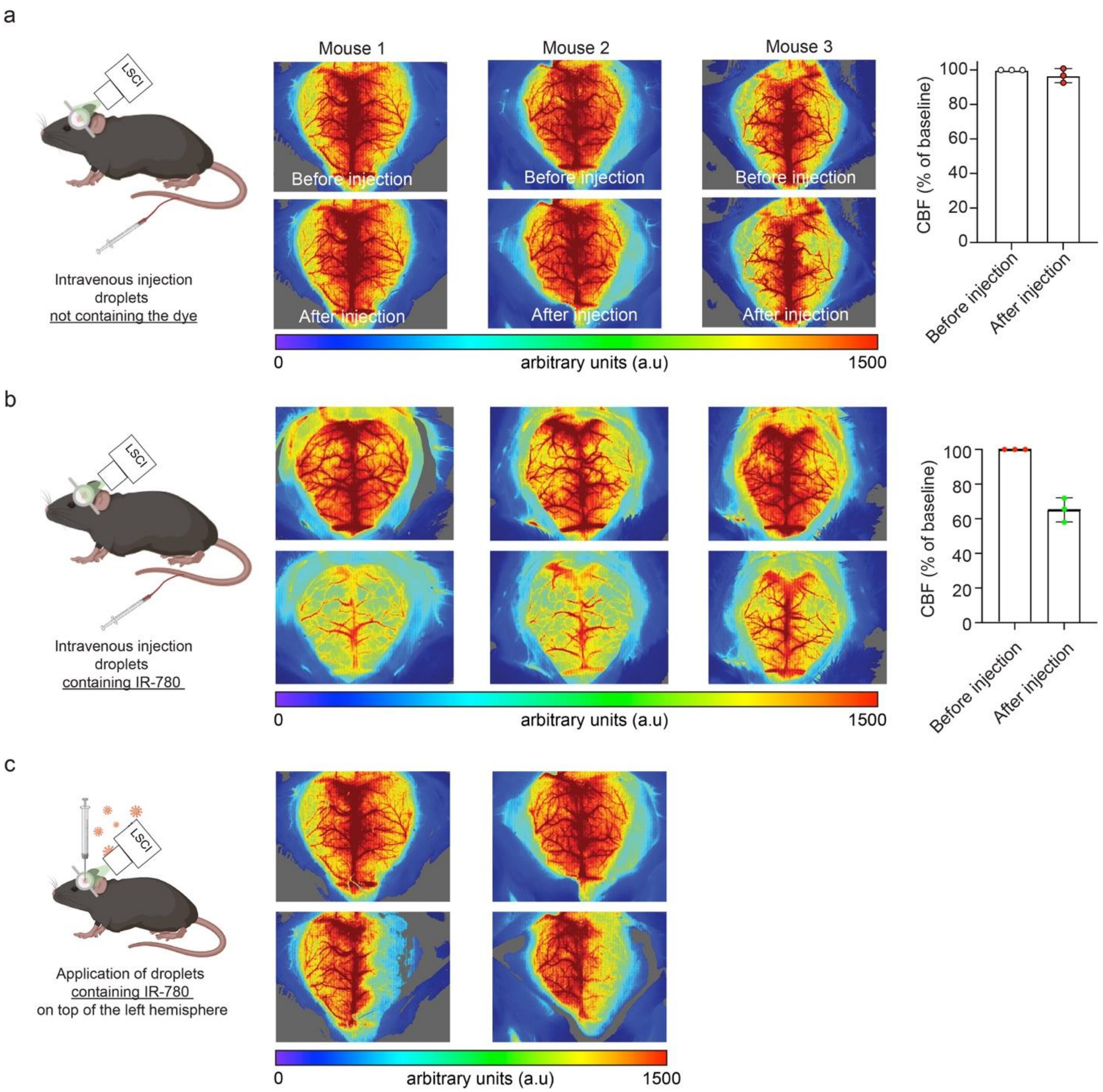

Supplementary Figure 10. Cortical perfusion assessment with laser speckle contrast imaging (LCSI). (a) LSCI image and quantification after intravenous injection of droplets not containing the dye. (b) LSCI image and quantification after intravenous injection of IR-780 droplets. (c) LSCI image taken after depositing ~20 μl of the emulsion of IR-780 droplets on top of the left hemisphere of the skull.

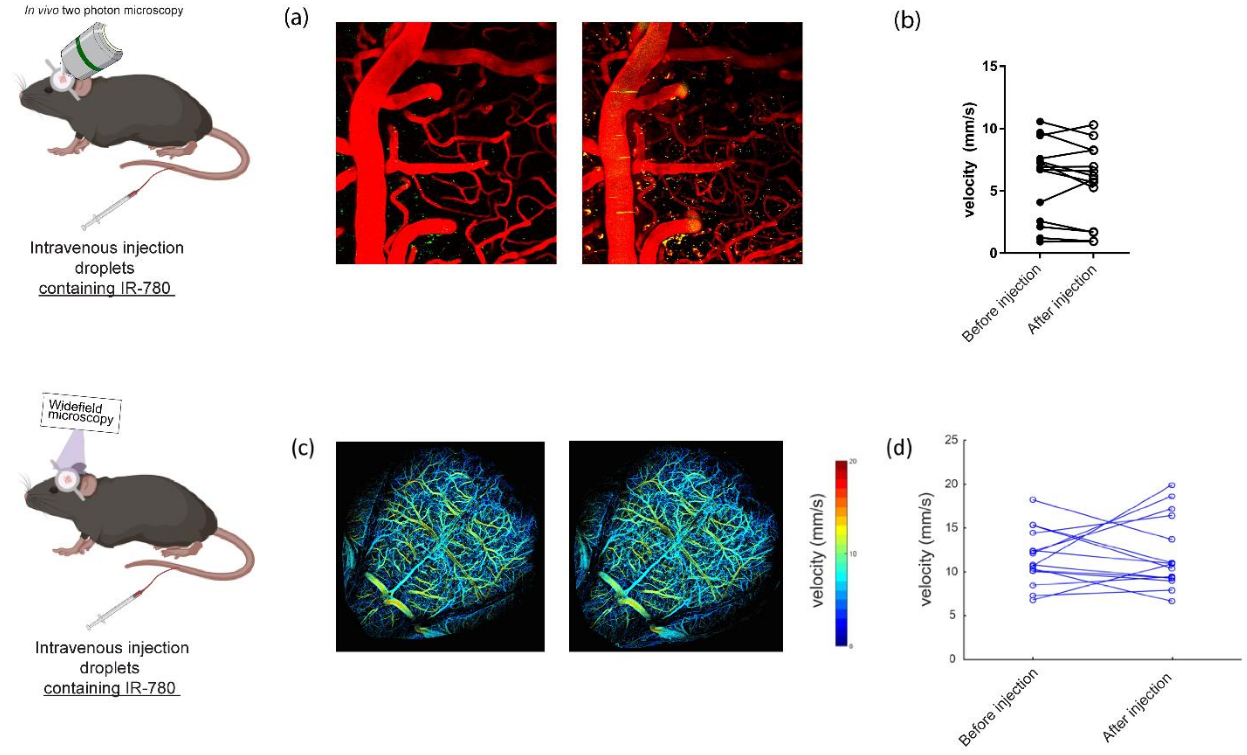

Supplementary Figure 11. Blood flow velocity quantification after microdroplet injection. (a) Two-photon images taken before and after injection of microdroplets. (b) Measured blood flow velocity at selected points before and after injection. No significant differences were observed (p=0.22 for a paired t-test). (c) Blood flow velocity maps acquired by tracking <5 μm fluorescent microbeads in the mouse cortex. (d) Measured blood flow velocity in vessels with a velocity range from 6-18 mm/s before and after injection. No significant differences were observed (p=0.72 for a paired t-test).

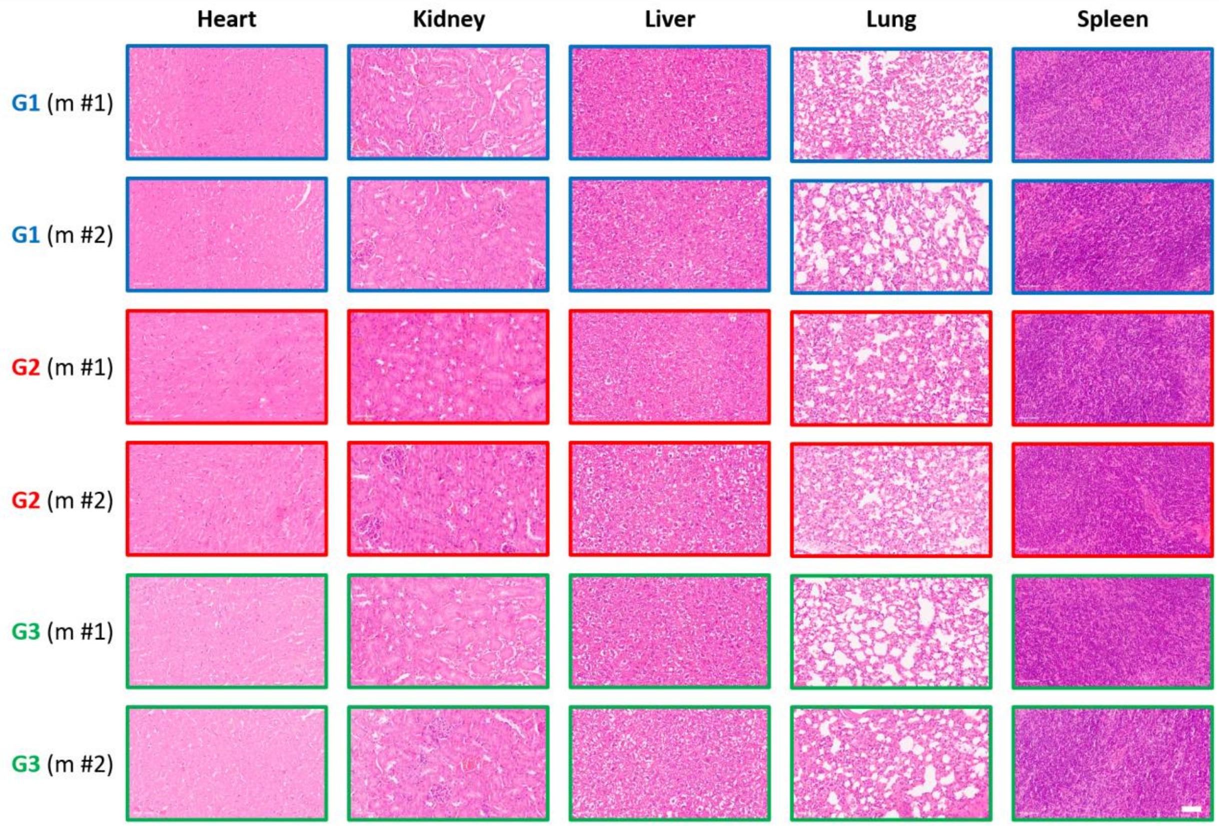

Supplementary Figure 12. H&E-stained histological images of major organs. Images with 40x magnification of the heart, kidney, liver, lung and spleen are shown. Sections were taken 14 days following injection of 100 µl of DCM-IR780 microdroplet emulsion (group G1, n=2 mice (m)), 100 µl of DCM microdroplet emulsion (group G2, n=2 mice (m)) and 100 µl of PBS (group G3, n=2 mice (m)). Scalebar – 60 µm.

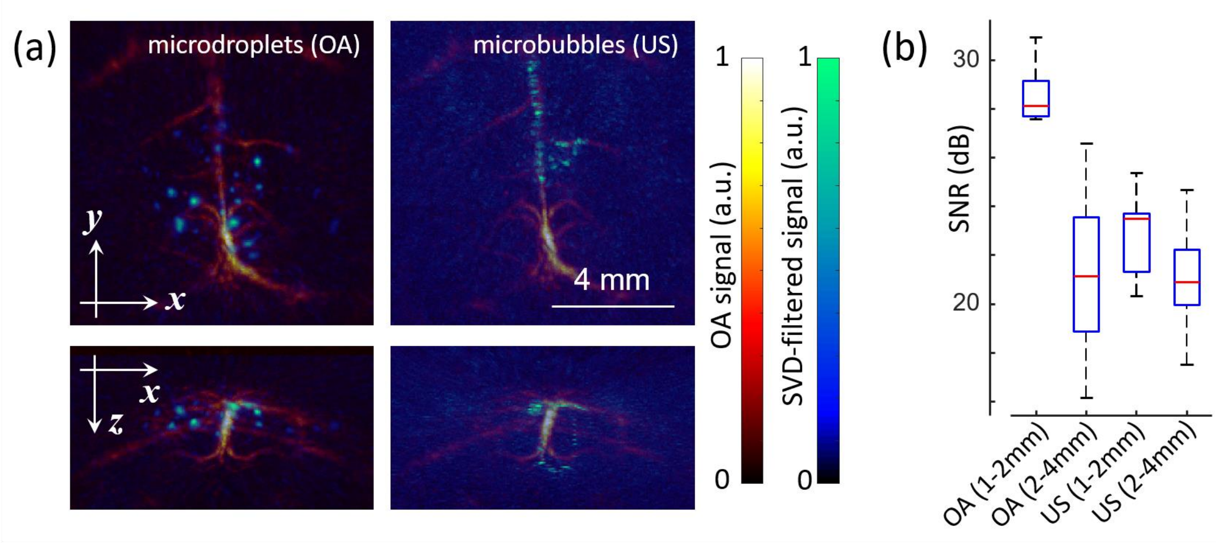

Supplementary Figure 13. Signal-to-noise ratio (SNR) of optoacoustic (OA) and pulse-echo ultrasound (US) images of microdroplets and microbubbles calculated via SVD filtering of a sequence of images. (a) OA images of microdroplets and US images of microbubbles superimposed to the OA image. (b) Boxplots calculated for 12 microdroplets/microbubbles for the same mouse. Maximum, upper quartile, median, lower quartile, and minimum values are shown. OA (1-2 mm) – 30.9 dB, 29.1 dB, 28.1 dB, 27.7 dB, 27.6 dB. OA (2-4 mm) – 26.6 dB, 23.6 dB, 21.1 dB, 18.9 dB, 16.2 dB. US (1-2 mm) – 25.4 dB, 23.7 dB, 23.5 dB, 21,3 dB, 20.3 dB. US (2-4 mm) – 24.7 dB, 22.2 dB, 20.9 dB, 19.9 dB, 17.5 dB.

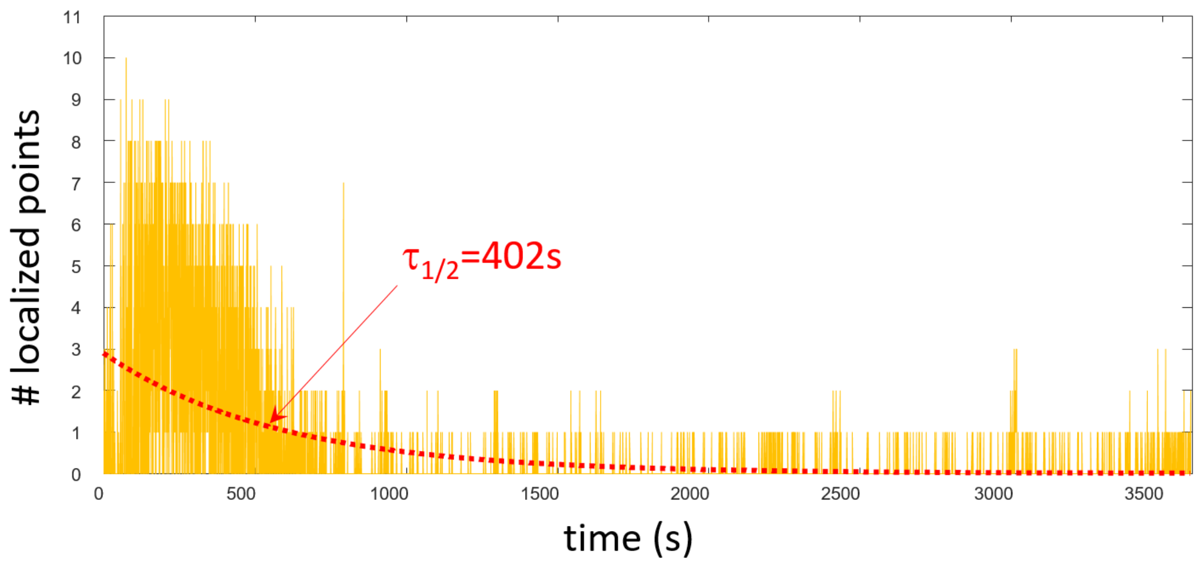

Supplementary Figure 14. Concentration of droplets as a function of time post injection. The number of localized points is plotted as a function of time for a sequence of frames acquired at 10 Hz for 1 hour. The fitted exponential curve is plotted in red, corresponding to a half-life of 402 s.

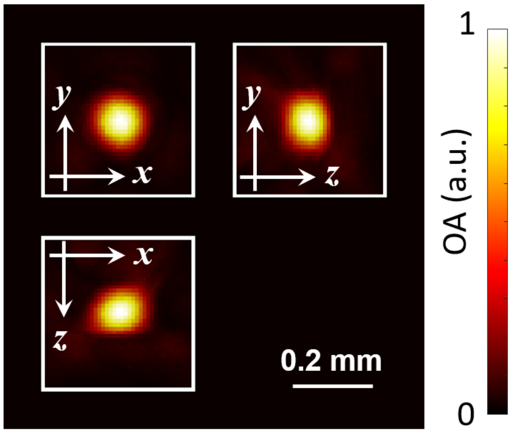

Supplementary Figure 15. Point spread function of the optoacoustic tomography (OAT) system. Maximun intensity projections (MIPs) along the three Cartesian coordinates are shown. It corresponds to the OAT image of a sub-resolution polyethylene microsphere with diameter ~30 μm.

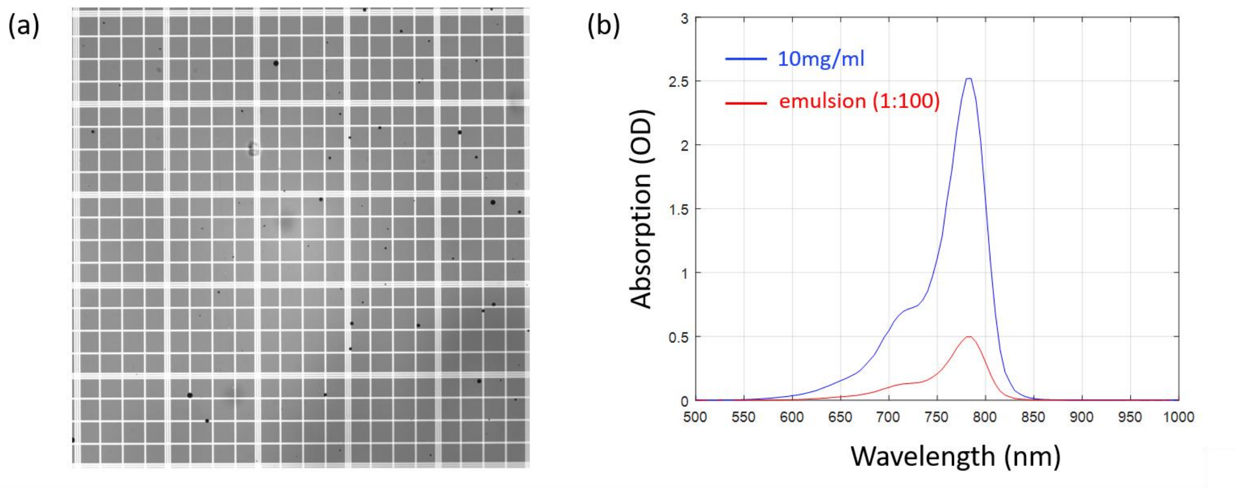

Supplementary Figure 16. Concentration of dichloromethane and IR-780. (a) Brightfield optical microscopy image of 9 μl of the emulsion containing the microdroplets. The size of the visible small squares in the Neubauer haemocytometeris is 50x50 μm^2. (b) Absorption spectrum of IR-780 in ethanol (10 mg/ml, blue) and of the microdroplet emulsion diluted 100 times in ethanol (red).

References

1 Wang, L. V. & Wu, H.-i. *Biomedical optics: principles and imaging*. (John Wiley & Sons, 2012).

2 Abramowitz, M., Stegun, I. A. & Romer, R. H. (American Association of Physics Teachers, 1988).

3 https://www.suva.ch/de-CH/material/Richtlinien-Gesetzestexte/grenzwerte-am-arbeitsplatz-aktuelle-werte.

4 Hellwig, A. Klinische Narkoseversuche mit Solaesthin. *Klinische Wochenschrift* **1**, 215-217 (1922).

5 Jiang, C. *et al.* Hydrophobic IR780 encapsulated in biodegradable human serum albumin nanoparticles for photothermal and photodynamic therapy. *Acta biomaterialia* **14**, 61-69 (2015).
