# Supplementary material for: Deep optoacoustic localization microangiography of ischemic stroke in mice
Source: Nat Commun. 2023 Jun 16;14:3584. doi: 10.1038/s41467-023-39069-1 (PMC10275987; doi:10.1038/s41467-023-39069-1)
Supplement: Supplementary file 3 — Description of Additional Supplementary Files [file 41467_2023_39069_MOESM3_ESM.pdf]

Description of Additional Supplementary Files

Supplementary Movie 1. Visualization of the flow of individual microdroplets (three-dimensional view). Differential optoacoustic (OA) image (green colormap) is superimposed to the OA image (orange colormap). Injection time point is indicated.

Supplementary Movie 2. Visualization of the flow of individual microdroplets (maximum intensity projections). Differential optoacoustic (OA) image (green colormap) is superimposed to the OA image (orange colormap). Injection time point is indicated.

Supplementary Movie 3. Localization optoacoustic tomography (LOT) image formation process. The rotating view of the localized points is shown.

Supplementary Movie 4. Comparison of optoacoustic tomography (OAT) and localization optoacoustic tomography (LOT). Rotating views are shown.

Supplementary Movie 5. Blood flow velocity map. The rotating view is shown.

Supplementary Movie 6. Oxygen saturation map of the mouse brain after stroke induction. The rotating view is shown.
