# Supplementary material for: Deep optoacoustic localization microangiography of ischemic stroke in mice
Source: Nat Commun. 2023 Jun 16;14:3584. doi: 10.1038/s41467-023-39069-1 (PMC10275987; doi:10.1038/s41467-023-39069-1)
Supplement: Supplementary file 11 — Reporting Summary [file 41467_2023_39069_MOESM11_ESM.pdf]

Corresponding author(s): Xose Luis Dean-Ben

Last updated by author(s): Mar 20, 2023

Reporting Summary

Nature Portfolio wishes to improve the reproducibility of the work that we publish. This form provides structure for consistency and transparency in reporting. For further information on Nature Portfolio policies, see our Editorial Policies and the Editorial Policy Checklist.

Statistics

For all statistical analyses, confirm that the following items are present in the figure legend, table legend, main text, or Methods section.

n/a	Confirmed	
☐	☒	The exact sample size (n) for each experimental group/condition, given as a discrete number and unit of measurement
☐	☒	A statement on whether measurements were taken from distinct samples or whether the same sample was measured repeatedly
☐	☒	The statistical test(s) used AND whether they are one- or two-sided<br>*Only common tests should be described solely by name; describe more complex techniques in the Methods section.*
☒	☐	A description of all covariates tested
☒	☐	A description of any assumptions or corrections, such as tests of normality and adjustment for multiple comparisons
☐	☒	A full description of the statistical parameters including central tendency (e.g. means) or other basic estimates (e.g. regression coefficient) AND variation (e.g. standard deviation) or associated estimates of uncertainty (e.g. confidence intervals)
☐	☒	For null hypothesis testing, the test statistic (e.g. F, t, r) with confidence intervals, effect sizes, degrees of freedom and P value noted<br>*Give P values as exact values whenever suitable.*
☒	☐	For Bayesian analysis, information on the choice of priors and Markov chain Monte Carlo settings
☒	☐	For hierarchical and complex designs, identification of the appropriate level for tests and full reporting of outcomes
☒	☐	Estimates of effect sizes (e.g. Cohen's d, Pearson's r), indicating how they were calculated

Our web collection on statistics for biologists contains articles on many of the points above.

Software and code

Policy information about availability of computer code

Data collection	Matlab R2019a. GPU-based OA reconstruction was implemented in openCL and executed in Matlab R2019a. LCSI images were acquired with MoorFLPI software V3.0. Two photon images were acquired with a customized version of ScanImage (r3.8.1; Janelia Research Campus).
Data analysis	Matlab R2019a. LCSI images were processed with MoorFLPI software. The acquired tif files in two-photon microscopy were analyzed with ImageJ software (NIH, version 1.41). Microdroplet tracking was performed with ©Jean-Yves Tinevez, 2019, wrapping matlab munkres algorithm implementation of ©Yi Cao 2009.

For manuscripts utilizing custom algorithms or software that are central to the research but not yet described in published literature, software must be made available to editors and reviewers. We strongly encourage code deposition in a community repository (e.g. GitHub). See the Nature Portfolio guidelines for submitting code & software for further information.

Data

Policy information about availability of data

All manuscripts must include a data availability statement. This statement should provide the following information, where applicable:

- Accession codes, unique identifiers, or web links for publicly available datasets
- A description of any restrictions on data availability
- For clinical datasets or third party data, please ensure that the statement adheres to our policy

The raw datasets before image reconstruction are too large to be publicly shared, yet they are available for research purposes from the corresponding author upon

request. The entire Allen Mouse Brain Atlas dataset and associated tools are available through an unrestricted web-based viewing application (http://mouse.brain-map.org).

Human research participants

Policy information about studies involving human research participants and Sex and Gender in Research.

Reporting on sex and gender	N/A
Population characteristics	N/A
Recruitment	N/A
Ethics oversight	N/A

Note that full information on the approval of the study protocol must also be provided in the manuscript.

Field-specific reporting

Please select the one below that is the best fit for your research. If you are not sure, read the appropriate sections before making your selection.

☒ Life sciences ☐ Behavioural & social sciences ☐ Ecological, evolutionary & environmental sciences

For a reference copy of the document with all sections, see nature.com/documents/nr-reporting-summary-flat.pdf

Life sciences study design

All studies must disclose on these points even when the disclosure is negative.

Sample size	The sample size was estimated according to the resources equation for an exploratory study.
Data exclusions	No mice were exluded in the study.
Replication	n=3 mice were used in LSCI and two-photon experiments to ensure reproducibility of the measured flow velocity values. n=2 mice were used in the histology analysis to ensure reproducibility of the results. The sizes of the droplets was also reproducibly measured.
Randomization	In the biosafety study, animals were randomly assigned to the three groups.
Blinding	Blinding is not applicable as there was no group allocation except for the biosafety study. In the biosafety study, physiological parameters with no bias were measured, thus no blinding was required.

Behavioural & social sciences study design

All studies must disclose on these points even when the disclosure is negative.

Study description	N/A
Research sample	N/A
Sampling strategy	N/A
Data collection	N/A
Timing	N/A
Data exclusions	N/A
Non-participation	N/A
Randomization	N/A

Ecological, evolutionary & environmental sciences study design

All studies must disclose on these points even when the disclosure is negative.

Study description	N/A
Research sample	N/A
Sampling strategy	N/A
Data collection	N/A
Timing and spatial scale	N/A
Data exclusions	N/A
Reproducibility	N/A
Randomization	N/A
Blinding	N/A

Did the study involve field work? ☐ Yes ☒ No

Reporting for specific materials, systems and methods

We require information from authors about some types of materials, experimental systems and methods used in many studies. Here, indicate whether each material, system or method listed is relevant to your study. If you are not sure if a list item applies to your research, read the appropriate section before selecting a response.

Materials & experimental systems

n/a	Involved in the study
☒	☐ Antibodies
☒	☐ Eukaryotic cell lines
☒	☐ Palaeontology and archaeology
☐	☒ Animals and other organisms
☒	☐ Clinical data
☒	☐ Dual use research of concern

Methods

n/a	Involved in the study
☒	☐ ChIP-seq
☒	☐ Flow cytometry
☒	☐ MRI-based neuroimaging

Animals and other research organisms

Policy information about studies involving animals; ARRIVE guidelines recommended for reporting animal research, and Sex and Gender in Research

Laboratory animals	Athymic nude-Fox1nu mice (n=5, 6-8 weeks old), BALB/c mice (n=14, 8-12 weeks old), and Swiss mice (n=12, 7 weeks old) were used in this study.
Wild animals	No wild animals were used in this study.
Reporting on sex	Sex of the animals was reported.
Field-collected samples	No field collected samples were used in the study.
Ethics oversight	All experiments except the biosafety study were approved by the Cantonal Veterinary Office Zürich (Switzerland). The biosafety study was approved by the Xunta de Galicia (Spain).

Note that full information on the approval of the study protocol must also be provided in the manuscript.
